# 639. Enhancing Transfer of Accountability in Burn Intensive Care Unit Nursing

**DOI:** 10.1093/jbcr/irag033.476

**Published:** 2026-04-06

**Authors:** Gavin Shantz

**Affiliations:** Sunnybrook Health Sciences Centre, Newmarket, ON

## Abstract

**Introduction:**

In a burn intensive care unit (ICU), effective communication during nurse handovers is critical to patient safety. Communication gaps during the transfer of accountability (TOA) are a root cause of communication breakdowns and contribute to preventable safety incidents. We designed a quality improvement (QI) initiative to standardize TOA between nurses that enhances the transfer of information during interprofessional rounds. Our SMART aim was to improve burn ICU nurses’ perception of a safe TOA culture by 20% and reduce monthly safety incidents by at least one-third.

**Methods:**

We conducted a two-pronged baseline assessment: TOA safety culture survey of 31 burn ICU nurses and a 3-month retrospective review of incident reports (baseline mean: 18 incidents/month). A burn-specific TOA tool was designed with frontline nurses, integrating established ICU handover best practices. A comprehensive education program accompanied the tool rollout. The intervention was implemented through eight weekly Plan-Do-Study-Act (PDSA) cycles (Dec 15, 2024 – Feb 15, 2025), allowing iterative refinement. Each cycle monitored outcome measures (safety incident rates, survey scores), process measures (TOA tool utilization, education attendance), and balancing measure (workflow impact). Run charts tracked safety incident trends, and qualitative nurse feedback was analyzed thematically, to guide adjustments.

**Results:**

Post-intervention, safety incidents decreased by 50% (from 18 to 9/month), and TOA-related safety culture scores improved by 20%, achieving both.

SMART objectives. Tool adherence exceeded 90% by the final cycle. Nurses reported improved clarity, reduced cognitive load, and enhanced interprofessional communication. No adverse workflow impacts were observed.

**Conclusions:**

A co-designed TOA tool, integrated with education and iterative PDSA refinement, significantly improved handover safety and reduced incidents in the burn ICU. This initiative provides a practical, scalable model for enhancing communication and safety culture in high-risk clinical settings.

**Applicability of Research to Practice:**

A burn ICU TOA tool, coupled with targeted education and iterative PDSA refinement, improved the process and outcomes of nurse handovers. This initiative reduced monthly safety incidents by 50% and enhanced nurses’ perceptions of a TOA safety culture at handover by 20%. Our approach provides a replicable framework for addressing communication gaps and underscores the importance of frontline staff engagement.

**Funding for the study:**

N/A.

